# A novel colonoscope with a wider field of view of 230° – first in human case

**DOI:** 10.1055/a-2524-5900

**Published:** 2025-02-11

**Authors:** Horst Neuhaus, Anh Nguyen, Johannes Grossmann

**Affiliations:** 1 Department of Gastroenterology, RKM740 Interdisciplinary Care Clinic, Düsseldorf, Germany

During colonoscopy, polyps can be missed, particularly those located behind folds or in tight bends, often due to the limited field of view (FOV) of standard colonoscopes 1 ( Fig. 1 ). A recent model-based study evaluating a novel colonoscope with a wider FOV of 230° (PENTAX Medical, Tokyo, Japan) showed a significant improvement in polyp detection compared to standard colonoscopes 2 ( Fig. 2 ). The video case highlights its first human use ( Video 1 ). The patient, a 69-year-old man with no comorbidities, was scheduled for surveillance after a polypectomy five years ago.

**Fig. 1:**
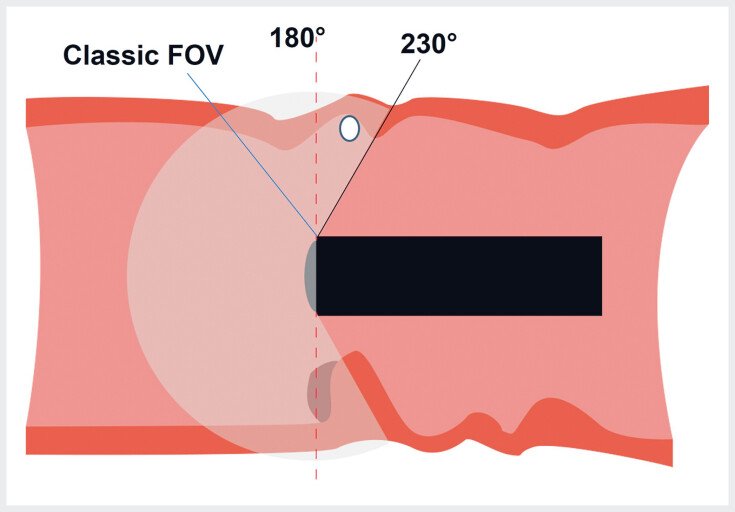
Different fields of view of an endoscope (not to scale).

**Fig. 2:**
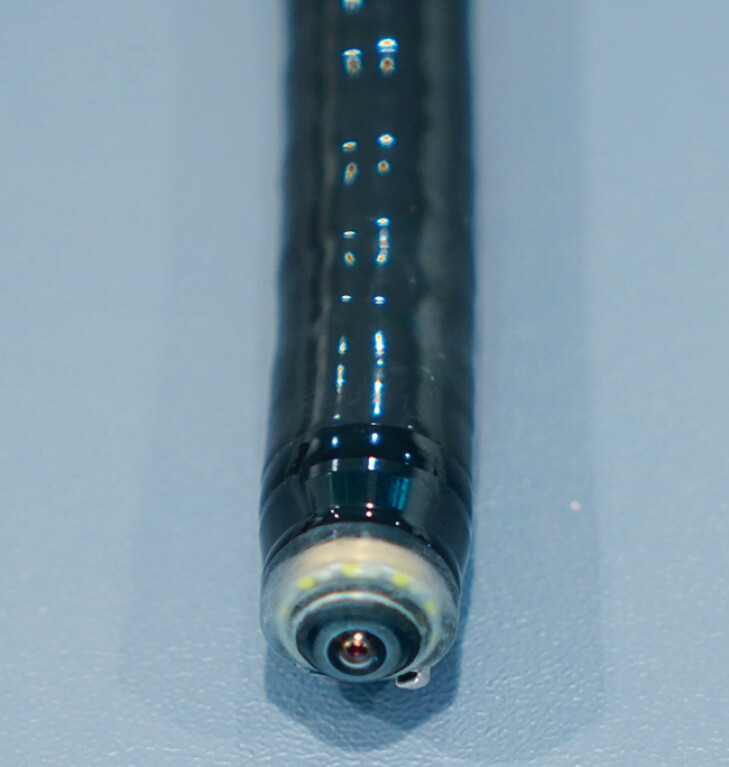
Close-up of the tip of the novel colonoscope.

First human use of novel colonoscope with a wider field of view of 230°.Video 1

The colonoscope was easily advanced into the cecum within three minutes. The wide FOV allowed an unlimited view of the cecum and facilitated the intubation of the terminal ileum ( Fig. 3 ). Withdrawal showed a flat lesion at the hepatic flexure. It was characterized as a NICE type 2 lesion after flushing with the integrated waterjet, enhanced imaging, and 1.5-fold electronic magnification. Cold snare resection could be easily performed. Two further small polyps located opposite each other were simultaneously captured in the transverse colon ( Video 1 ). Cold snare resection was performed and the specimens were captured by suction. Further withdrawal allowed a complete evaluation of the left colon despite diverticula without major movement of the endoscope tip ( Fig. 4 ). The sigmoid was subsequently inspected with a standard colonoscope, suggesting that complete visualization is more challenging due to the smaller FOV. Histology of the resected polyps revealed low grade tubular adenomas.

**Fig. 3:**
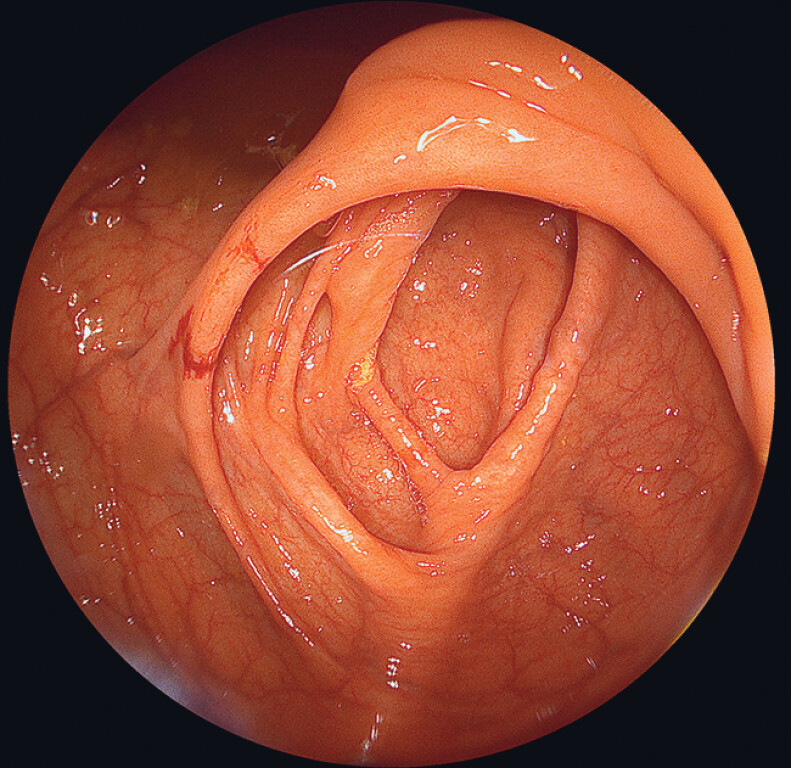
View of the cecum and ileocecal valve.

**Fig. 4:**
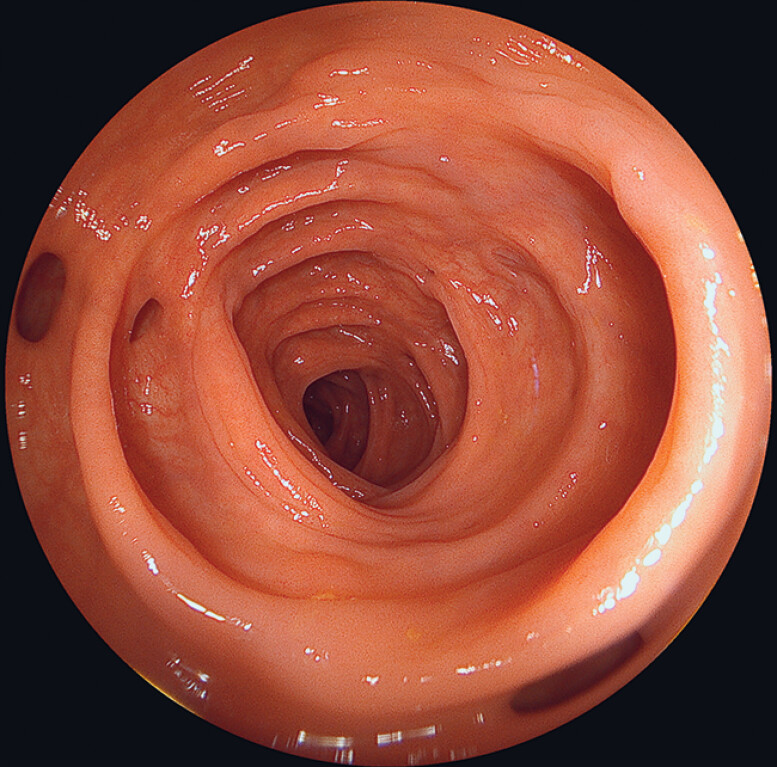
Complete visualization of the sigmoid circumference.

The case suggests that the new colonoscope is easy to use and facilitates visualization of the entire colorectal circumference. The image quality is excellent. Air insufflation, lens irrigation, and suction quality are comparable to standard colonoscopes. It proves highly effective in the detection and removal of small polyps and flat lesions, including those concealed behind folds.

Endoscopy_UCTN_Code_TTT_1AQ_2AB

## Notes

Endoscopy E-Videos https://eref.thieme.de/e-videos
*E-Videos* is an open access online section of the journal *Endoscopy* , reporting on interesting cases and new techniques in gastroenterological endoscopy. All papers include a high-quality video and are published with a Creative Commons CC-BY license. Endoscopy E-Videos qualify for HINARI discounts and waivers and eligibility is automatically checked during the submission process. We grant 100% waivers to articles whose corresponding authors are based in Group A countries and 50% waivers to those who are based in Group B countries as classified by Research4Life (see: https://www.research4life.org/access/eligibility/ ). This section has its own submission website at https://mc.manuscriptcentral.com/e-videos .
